# Hemocytes in the extrapallial space of *Pinctada fucata* are involved in immunity and biomineralization

**DOI:** 10.1038/s41598-018-22961-y

**Published:** 2018-03-15

**Authors:** Jingliang Huang, Shiguo Li, Yangjia Liu, Chuang Liu, Liping Xie, Rongqing Zhang

**Affiliations:** 1 MOE Key Laboratory of Protein Sciences, School of Life Sciences, Tsinghua University, Beijing, 100084 China; 2 Tsinghua-Peking Joint Center for Life Sciences, School of Life Sciences, Tsinghua University, Beijing, 100084 China; 3 Department of Biotechnology and Biomedicine, Yangtze Delta Region Institute of Tsinghua University, Jiaxing, 314000 China

## Abstract

In bivalves, the mantle tissue secretes organic matrix and inorganic ions into the extrapallial space (EPS) to form the shells. In addition, more and more evidences indicate the participation of hemocytes in shell mineralization, but no direct evidence has been reported that verifies the presence of hemocytes in the EPS, and their exact roles in biomineralization remain uncertain. Here, we identified hemocytes from the EPS of *Pinctada fucata*. Numerous components involved in cellular and humoral immunity were identified by proteome analysis, together with several proteins involved in calcium metabolism. The hemocytes exerted active phagocytosis and significantly upregulated the expression of immune genes after immune stimulation. A group of granulocytes were found to contain numerous calcium-rich vesicles and crystals, which serve as a calcium pool. During shell regeneration, some genes involved in calcium metabolism are upregulated. Strikingly, most of the shell matrix proteins were absent in the hemocytes, suggesting that they might not be solely responsible for directing the growth of the shell. Taken together, our results provided comprehensive information about the function of hemocytes in immunity and shell formation.

## Introduction

Bivalves are among the most successful invertebrates living in a broad range of aquatic habitats. This is partially due to their thin but rigid shell, which is the first line of defense against pathogens in an ambient environment and an efficient protection from predators. Bivalve shells are mainly composed of calcium carbonate (>95%) and a framework of organic matrix (<5%). Low in content, the organic matrix reinforces the shell 3000 times over the monolithic CaCO_3_^1^. The formation of the shell is rather complex and precisely controlled^2,3^. According to the classical theory, mantle tissue plays a central role in shell formation. It is believed that the shell is precipitated in the extrapallial space (EPS), an external cavity enclosed by the mature shell and the mantle tissue. The cells lining the outer surface of the mantle tissue secrete organic materials such as shell matrix proteins (SMP) and inorganic ions into the EPS, thus forming the extrapallial fluid (EPF). The EPF can provide a suitable and elaborately controlled microenvironment for SMP to self-assemble into a framework, which subsequently promotes precipitation of the calcium carbonate. In the past decades, numerous SMPs were identified and shown to function in polymorph selection, orientation, nucleation and the growth of crystals^4^.

In addition to the widely recognized mantle-mediated calcification theory, biomineralization mediated by hemocytes was recently noted. Mount AS, *et al*.^5^ proposed that in the eastern oyster, a special type of refractive hemocyte could mediate the calcification process and respond to shell damage. These hemocytes seemed to fill the vacancy of the large non-exchangeable calcium pool left by mantle tissue. Furthermore, hemocytes containing Ca-positive granular contents and CaCO_3_ crystals participate in shell regeneration in oysters^6^ and mussels^7^, indicating that hemocytes may play a role in shell formation. Hemocytes in some oysters seemed to contribute to part of the SMP secretion^8^, leading to further studies on the exact role that hemocytes play during shell formation. Unfortunately, in the literature, bivalve hemocytes were empirically postulated to be present in the EPS, where biomineralization takes place, leaving the verification and origin of hemocytes in this external cavity overlooked. Although hemocytes in many mollusks can precipitate crystals in the cytoplasm^9,10^, the definite function related to biomineralization and the mechanism of these phenomena are not well understood. Additionally, it is well known that hemocytes are the main immune effector in mollusks and may be involved in many other physiological events, such as nutrient transportation, detoxification and wound healing^5,11^. How hemocytes fulfil their roles in biomineralization in addition to these arduous tasks should be analyzed.

In the past decades, shell formation in the pearl oyster *P inctada fucata* has been studied extensively, not only because it is the most important species for seawater-cultured pearls, but also for the potential template in bioinspired ceramics. The published draft genome and transcriptome data of *P. fucata* facilitates in-depth studies on the genetic and molecular regulation of the biomineralization process^12,13^. In previous studies, our group successfully identified hemocytes from *P. fucata* and showed their potential roles in shell regeneration^6^. To further analyse their physiological features during shell formation, we studied the hemocytes in the EPS of *P. fucata*. We demonstrated that these hemocytes in the EPS originate from the circulation system, and their comprehensive functions in immunity and biomineralization were investigated on multiple levels.

## Results

### Morphology of hemocytes in the EPS

**Figure 1:**
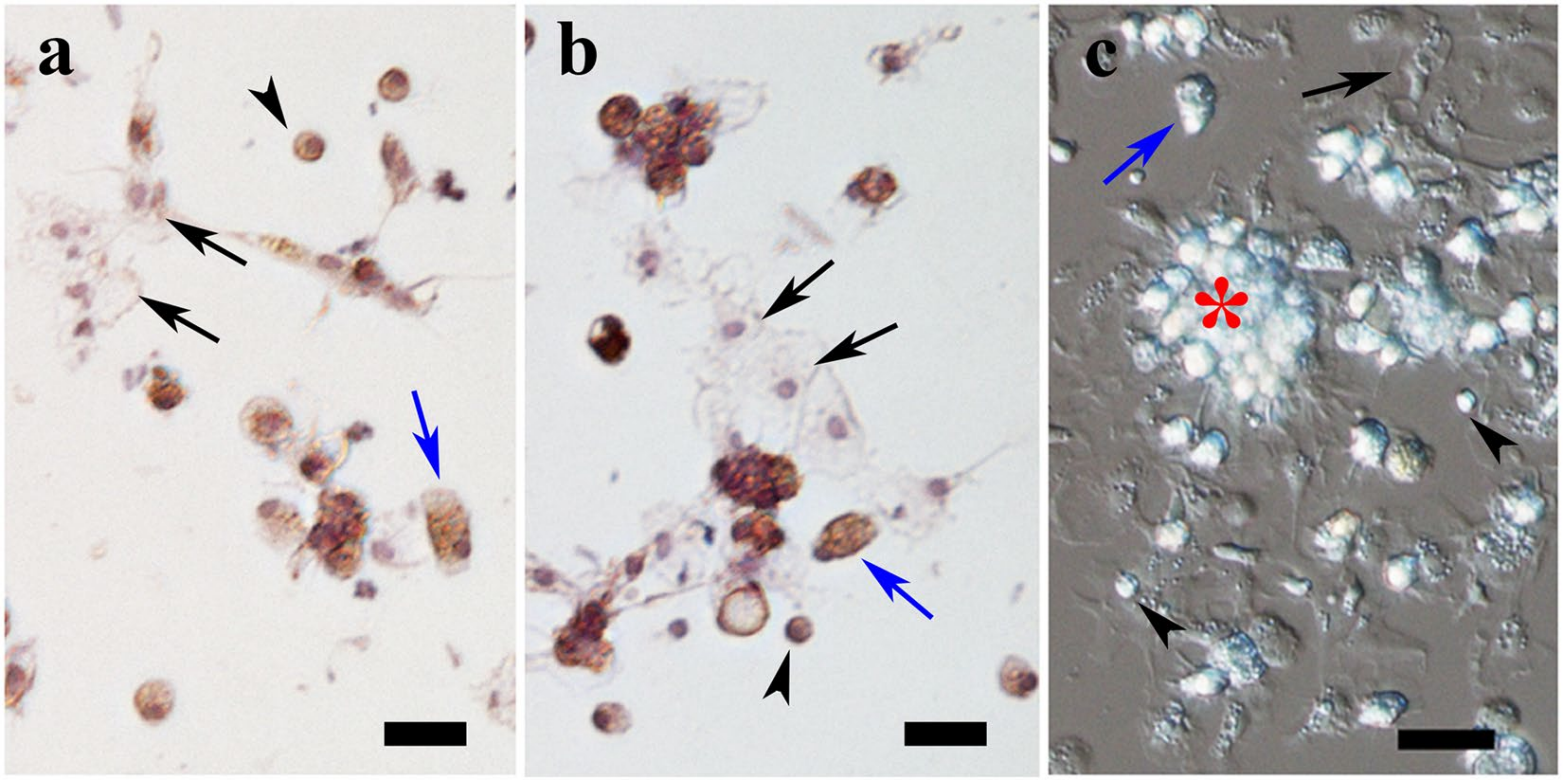
Comparison of hemocytes from EPS and adductor muscle in *P. fucata*. a and b, H&E stain of the different hemocyte cell types from EPS (**a**) and adductor muscle (**b**). (**c**) Cell clumps (red star) formed by both granulocytes and hyalinocytes from EPS, DIC image. In a, b and c, black arrows represent hyalinocytes, blue arrows represent granulocytes, and the black arrow heads represent blast-like cells. Bar = 20 µm.

To verify that the cells in the EPS were hemocytes, we first examined their characteristics and morphology. As shown in Fig. 1a and b, the morphologies of cells from the EPS and adductor muscle sinuses were identical, reminiscent of the hemocytes in *P. fucata* reported by Shiguo Li *et al*.^14^. Hyalinocytes and granulocytes were prominent in the extracted EPF, together with the blast-like cells. For the hyalinocytes, the cytoplasm was transparent with several pseudopodia-like synapses formed at the marginal region, while the nucleus was clear. Granulocytes could be identified by the pink eosin cytoplasm, and the granules in the cytoplasm refracted light (Fig. 1c).When exposed *in vitro*, these hemocytes in the EPS tended to agglutinate and adhere to the bottom, consistent with those reported in other bivalves^15^.

### Fluorescent tracking of hemocytes in the EPS

The origin of the hemocytes in the EPS was tracked by labelled cells. When injected into the adductor muscles sinuses, the labelled cells were detected in the EPS after 24 h (Table 1). Hemolymph extracted from adductor muscles also contained labeled cells as expected. Meanwhile, labelled cells were detected in all of the tested sites when the EPS was injected with labelled cells, though the detected labelled cells varied in numbers.

**Table 1:** Tracking hemocytes between the EPS and the circulation system.

Labelled cells	Percent	
	EPS	AMS
EPS injection	3.41	2.46
AMS injection	4.75	5.68

Cell samples were extracted and examined from the EPS and adductor muscle sinuses. The percentages of labelled hemocytes in the overall cell numbers were calculated. The results represent four duplicates.

### Proteomic analysis

When SDS-PAGE was performed, the protein bands were similar between the hemocyte cellular proteins from the EPF and adductor muscle (Supplementary Figure 1). Using LC-MS/MS method, a large amount of cellular proteins was identified. First, two molecular markers for hemocytes in invertebrates, namely Filamin-C and copper/zinc superoxide dismutase (SOD), were identified. The former is one of the most abundant fragments in the proteome. Some proteins were identified and grouped into three categories, namely cellular immunity, humoral immunity and calcification (Table 2, Supplementary Tables 2–4). The conceptions of cellular and humoral immunity were employed based on well-acknowledged criteria^16^. In the cellular immunity category, the members were found to mediate phagocytosis and encapsulation, including hemocyte agglutination, migration, endocytosis and degradation. In the humoral immunity category, components involved in reactive oxygen species (ROS) production and metabolism were identified, together with several proteinases and antibacterial factors. In the third group, we identified proteins closely related to calcium metabolism, such as calcium-transporting ATPase, calponin-2, calponin-3, and calmodulin. Interestingly, ACCBP, mantle gene 11, mantle gene 4 and Pif were also present in the proteome, though the scores were relatively low compared to other cellular components.

**Table 2:** Components with partially known functions in the proteome of hemocytes in the EPS.

Description	Score	Function	Reference
**Cellular immunity**			
Galectin	1090.78	Agglutination, pathogen recognition	^40,47^
Allograft inflammatory factor-1	374.42	Inflammation, wound healing	^31,41,48^
Apoptosis-inducing factor 3	341.21	Apoptosis	^49^
**Humoral immunity**			
Copper/zinc superoxide dismutase (Fragment)	324.38	ROS removal, detoxification	^25,50^
Dual oxidase 2, partial	199.07	ROS generation	^37^
Metalloendopeptidase	187.82	Inflammatory response, proliferation	^51^
Peroxiredoxin	157.23	ROS removal	^25^
Alpha-2-macroglobulin	31.04	Blood clotting, protease inhibitor	^42^
**Calcification-related**			
Calreticulin	1170.37	Calcium transport and storage	^52^
Calponin-3 (Fragment)	621.53	Calcium-binding, actin-binding	^13,53^
Calponin-2 (Fragment)	228.80	Calcium-binding, actin-binding	^13,53^
Calmodulin	164.57	Calcium metabolism regulator	^54^
Carbonic anhydrase-related protein VIII	155.21	Calcification, bone resorption	^18,55^
Mantle protein 11	49.94	Unclear	^43^

### Phagocytosis of yeast

**Figure 2:**
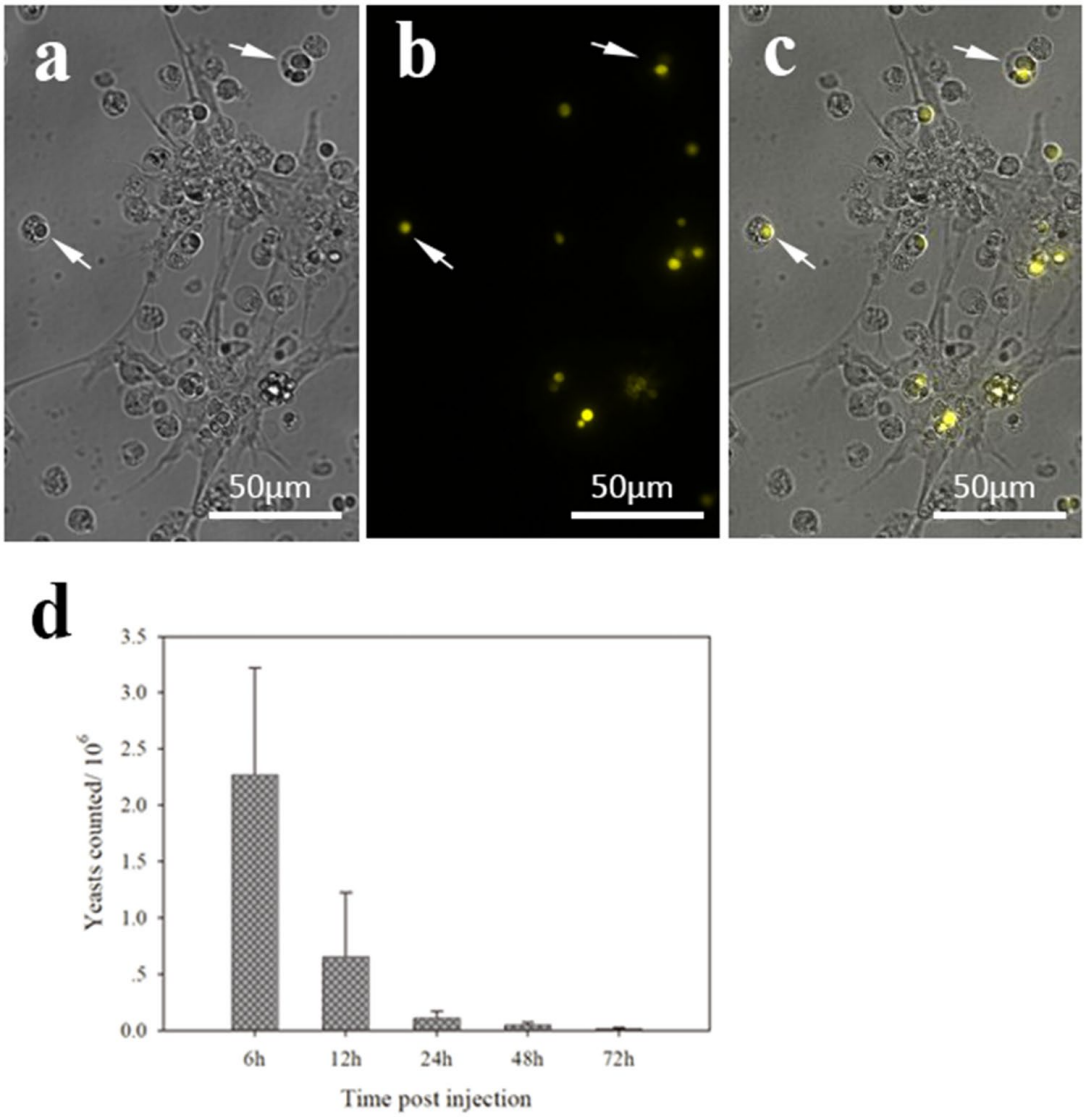
Phagocytosis of yeast by hemocytes in EPS. (**a**) Bright field; (**b**) YFP channel (542 nm); (**c**) merged picture of a and b. Some yeast cells with YFP signal were present inside the hemocytes (white arrows). (**d**) Time series of the residual yeast in the EPS. The data represent an average of 3–5 replicates.

Phagocytosis by hemocytes is a principal way to clear pathogens in mollusks. As shown in Fig. 2a–c, many hemocytes with at least one yeast cell inside the cytoplasm were observed when the EPS was filled with injected yeasts. While granulocytes were the main cell type undergoing phagocytosis, hyalinocytes were seldom found to contain any labelled yeasts in the cytoplasm. The hemocytes underwent efficient phagocytosis and clear most of the yeast in 24 hours (Fig. 2d).

### Crystal-bearing granulocytes in the EPS

**Figure 3:**
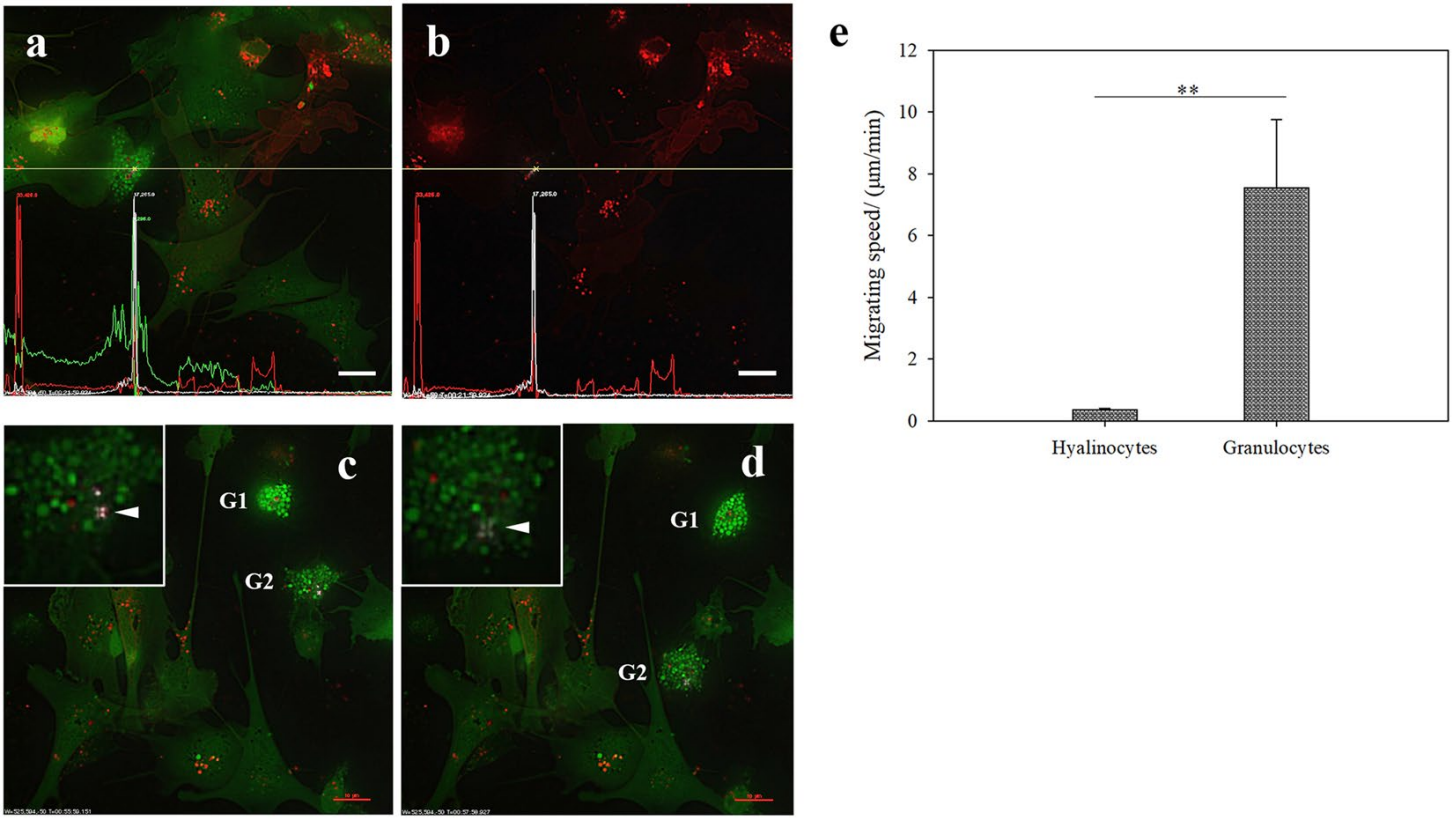
Crystal-bearing granulocytes in the EPS of *P. fucata*. (**a**) Micrograph of the transparent hyalinocytes and the crystal-bearing granulocytes in different channels, 488 nm for calcein-AM, 542 nm for DiI and polar light for the crystal. The signal intensity distribution across the four-leaf clover spot was shown on the bottom. (**b**) Merged micrograph of 542 nm and polar light channels. (**c** and **d**) Timing diagrams of two migrating granulocytes (G1 and G2). Inset in c and d, magnification of G2 granulocyte, showing the birefringent particle in the granulocyte (white arrow head). c corresponds to 00: 56 min, and d corresponds to 00:58 min in the DeltaVision raw data. Scale bar = 10 µm. (**e**) Migrating speed of hyalinocytes and granulocytes in the EPS. The results represent the average of six replicates.

A special type of granulocyte was found to contain crystals in the EPS. Inside the cytoplasm there were many vesicles containing high concentrations of calcium, compared to the hyalinocytes nearby (Fig. 3a). Interestingly, several birefringent particles were visible in these granulocytes. The peaks of DiI and polar light signals co-localized in the same granulocyte (Fig. 3b). Another feature of the granulocytes was rapid mobility. In Fig. 3c and d, two granulocytes migrated at a high speed in between the hyalinocytes, one of which was accompanied by a four-leaf clover spot during the migration. These granulocytes could migrate at a speed of 7.54 µm/min on average, while the hyalinocytes barely moved (Fig. 3e).

### Gene expression after LPS and notching stimulation

**Figure 4:**
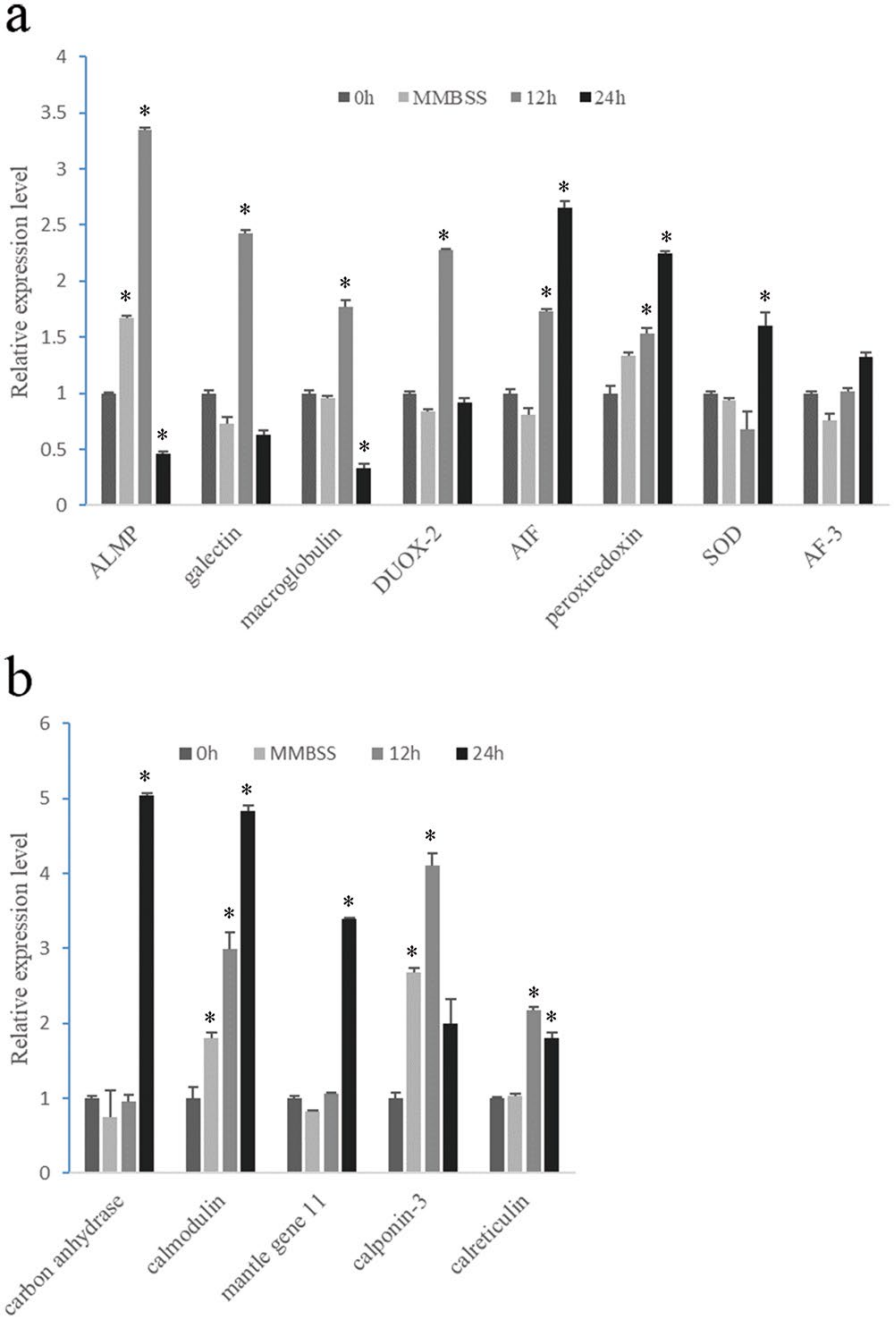
Gene expression of hemocytes in the EPS after stimulation with LPS. Genes involved in immunity (**a**) and the calcification process (**b**) were determined by RT-PCR. ALMP, astacin-like metalloproteinase; DUOX-2, dual oxidase 2; AIF, Allograft inflammatory factor; SOD, superoxide dismutase; AF-3, apoptosis induced factor 3. (P < 0.05).

Eight genes related to cellular immunity and humoral immunity, and six genes related to calcification were tested during LPS and notching stimulation. Most of the tested immune genes responded to LPS injection (Fig. 4a). ALMP, galectin, and DUOX-2 were upregulated at 12 h and declined substantially. AIF, peroxiredoxin and SOD were significantly upregulated after 24 h, showing a lagging response pattern, while apoptosis-induced factor 3 showed no response. Alternatively, genes involved in calcification also responded but were in a different mode (Fig. 4b). Carbonic anhydrase-related protein VIII, calmodulin, and mantle gene 11 reached their highest expression level at 24 h, while calponin-3 and calreticulin displayed a faster response.

**Figure 5:**
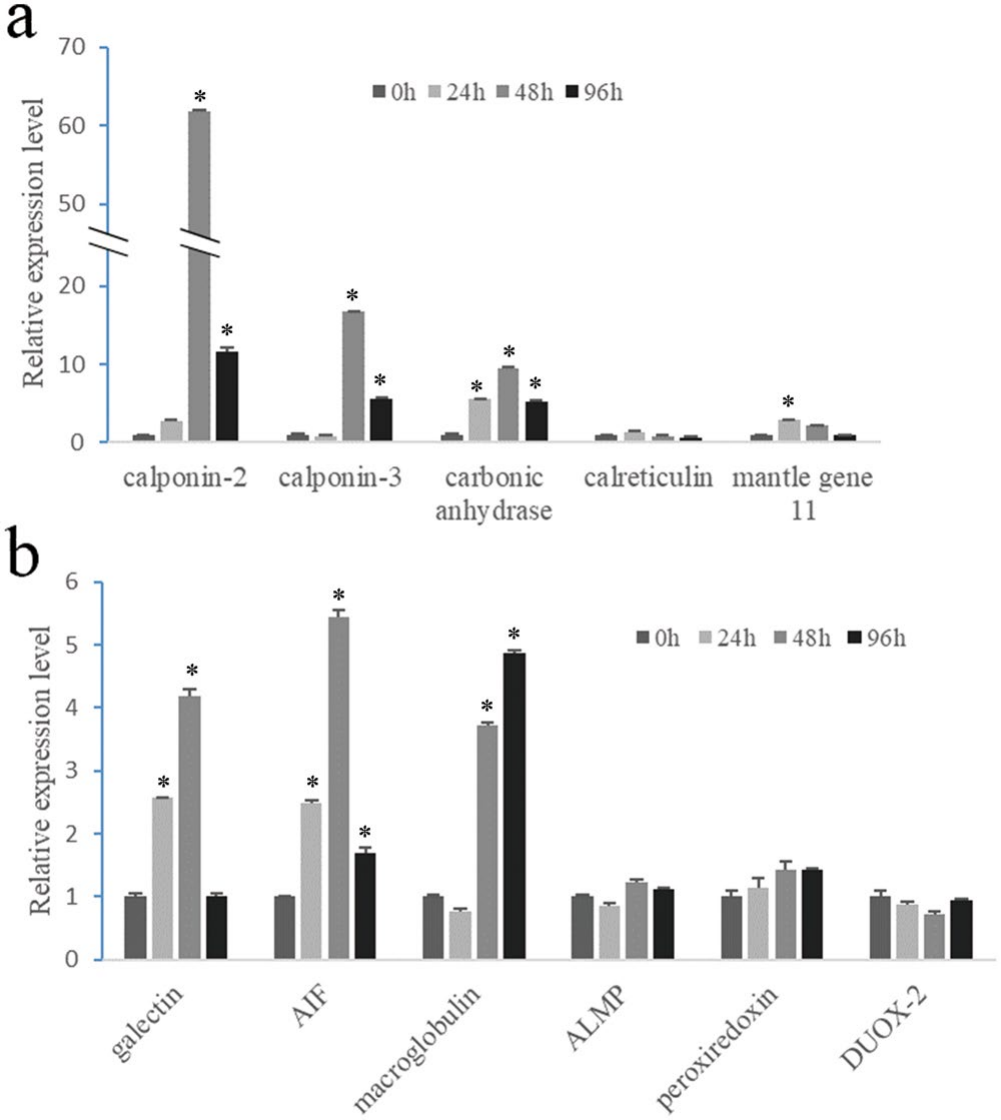
Gene expression of hemocytes in the EPS during shell regeneration. Genes involved in immunity (**a**) and the calcification process (**b**) were determined by RT-PCR. ALMP, astacin-like metalloproteinase; DUOX-2, dual oxidase 2; AIF, Allograft inflammatory factor. (P < 0.05).

Generally, oysters begin shell regeneration in several hours^17^, and a newly formed prism could be seen as early as 96 h after shell notching in this study. As a result, upregulation of expression levels was detected for carbonic anhydrase-related protein VIII (62-fold), calponin-2 (17-fold) and calponin-3 (10-fold) within 48 h post notching and maintained high expression after 96 h (Fig. 5a). Calreticulin and mantle gene 11 showed relatively small fluctuations. Furthermore, some of the immune genes, including ALMP, AIF and macroglobulin, also showed significant upregulation (Fig. 5b).

### Comparison of gene expression between mantle and hemocytes

**Figure 6:**
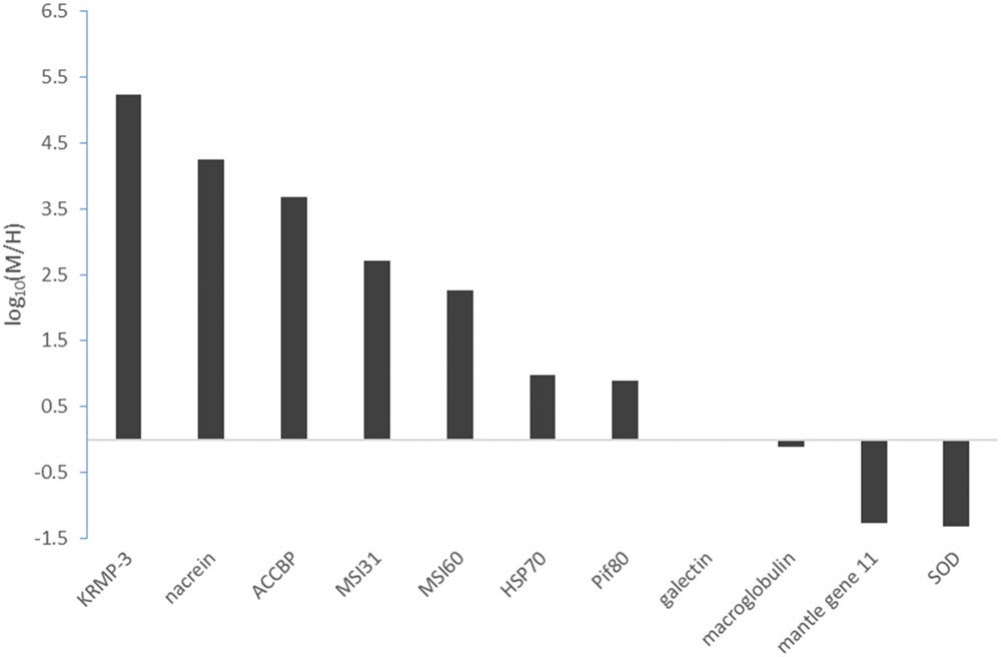
Comparison of gene expression between hemocytes in the EPS and mantle. Eleven genes were selected, including seven genes participating in the calcification process and four participating in the immune response. The upper and lower panels indicate that the gene expression levels in the mantle were higher or lower than those in the hemocytes, respectively. M, mantle; H, hemocytes. KRMP-3, lysine-rich matrix protein 3; ACCBP, amorphous calcium carbonate binding protein; HSP70, heat shock protein 70; SOD, superoxide dismutase.

To explore the ability of hemocytes to secrete SMP, we tested the expression of important SMP genes in the mantle and hemocytes, as well as some immune genes. As shown in Fig. 6 and Supplementary Table 5, most of the tested genes varied dramatically between the two samples. SMPs including KRMP3 and MSI31 from prism, and MSI60 from nacre, or nacrein and Pif80 from both shell layers were mainly expressed in the mantle, consistent with previous reports^18–20^. Meanwhile, SOD, mantle gene 11 and macroglobulin were enriched in hemocytes.

## Discussion

In addition to their central roles in innate immunity, the idea that hemocytes participate in the biomineralization process has recently emerged. However, some fundamental questions remain. First, hemocytes must reach the biomineralization front, the external cavity EPS. In this study, we validated the perception that the free-living cells in the EPS were hemocytes, and the morphological features and cellular proteins were identical to those hemocytes preserved inside the body (Fig. 1, Supplementary File 1). This finding was further affirmed by two molecular makers (Filamin-C and SOD) for hemocytes in invertebrates^21,22^, which were detected by proteomic analysis and real-time PCR.

The origin of hemocytes in the EPS was traced by labelled cells. The hemocytes circulated between the EPS and vessels. How the hemocytes migrated out from the circulation system was unclear. In some bivalves, hemocytes were reported to penetrate tissues and epithelium layers and to wander in some regions of the body^23,24^. Li, Shiguo, *et al*.^6^ showed that there were some secretory cavities on the outer surface of the mantle, and some hemocyte-like cells could be seen nearby. It is likely that the hemocytes passed through the mantle epithelium layer and migrated into the EPS through the secretory cavities. Meanwhile, the hemocytes in the EPS could rejoin the circulation through the cavities. Moreover, the tissue-infiltrating feature would give hemocytes a broader action to kill invasive microbes.

Bivalves do not possess adaptive immunity, and the innate immunity has been extensively expanded for adaptation^25^. Nevertheless, concepts of cellular and humoral immunity are applied to depict the immune responses in mollusks, based on the requirement of intact hemocyte participants^16^. Many proteins were involved in cellular or humoral immunity in our proteome data. Among them, galectins, C-type and F-type lectins participate in non-self recognition^26,27^. A certain amount of microbes was present in the EPS (Supplementary Figure 2), indicating that pathogens would occasionally infiltrate the EPS from ambient sea-water. When pathogens were recognized, phagocytes are activated and recruited to clear the pathogens. Phagocytosis and encapsulation are the prominent defensive mechanisms of cellular immunity in mollusks. YFP-bearing yeasts, mimicking pathogens that invaded into the EPS, were eliminated by hemocytes as expected (Fig. 2). Although phagocytosis by hyalinocytes was reported^28^, our data showed that only the granulocytes but not the hyalinocytes exhibited phagocytic activity, consistent with previous studies^29,30^. Furthermore, numerous proteins involved in hemocyte migration and promoting phagocytosis were identified (Supplementary Table 2), including transgelin, integrin and allograft inflammatory factor^31–33^. Cathepsin family members and metalloendopeptidase degrade exogenous proteins of pathogens in the cytoplasm^34^. Some members of NF-kappa B, IFN and TNF signaling pathways were present, and these pathways are believed to regulate immune responses^35^.

ROS, corresponding to humoral immunity components, could form biocidal products with nitrous oxides, thus killing of microbes^36^. DUOX-2, responsible for ROS production^37^, was abundant in hemocytes and was upregulated significantly post LPS induction. By producing generous ROS, together with other antibacterial components (Supplementary Table 3), oysters can kill the invading microbes. When the pathogens were cleared, the excess ROS should be removed to avoid poisoning the oysters themselves. This was accomplished by both enzymatic antixidants, such as catalase and SOD^25^, and redox components, such as thioredoxin, peroxiredoxin, and GSH, which were all detected in hemocytes, suggesting that defense mechanisms against pathogens were conserved among marine bivalves.

The enriched nutrients (proteins and polysaccharides) in the EPF were favorable for the exogenous intruders like microbes to burst. Incontrollable rapid proliferation of bacteria in the EPS led to abnormal shell deposition or even death in clams and oysters^38,39^. Therefore, the hemocytes are crucial for maintaining homeostasis in the EPS by clearing the intruding pathogens by applying cellular and humoral immunity, thus ensuring the shell formation process. Moreover, the hemocytes in both the EPS and circulatory system showed fast responses to the LPS stimulation (Fig. 4a and Supplementary Figure 3), indicating a finely control of the homeostasis in the EPS by the oyster. Interestingly, some slightly differences have been observed. Said that, the hemocytes in the circulatory system are more sensitive to the injected marine molluscan balanced salt solution (MMBSS) which might cause fluctuation in the volume of body fluids. We don’t know if this phenomenon is relevant in bivalves.

Calcium supply is a vital constraint during CaCO_3_ precipitation, especially during shell regeneration. We speculated that the hemocytes provide a replenishing pathway for the calcium supply. In mussels^7^ and oysters^6^, crystal-carrying hemocytes were reported and presumed to participate in shell regeneration. Indeed, we found that some granulocytes in *P. fucata* carried vesicles containing concentrated calcium and crystals (Fig. 3). Although the exact composition of these crystals was unclear, it might be calcium carbonate according to our previous study^6^. Moreover, some genes involved in calcium metabolism were upregulated dramatically post notching and LPS stimuli (Figs 4b and 5a), and the response pattern was similar to PfN44, Nacrein and KRMP in mantle tissues during shell regeneration^17^. These results suggest that the hemocytes might assist the mantle tissue in shell regeneration by accelerating the calcium transportation when stressors were detected. Interestingly, the injurious notching operation also activated some immune genes, for example, galectin, AIF and macroglobulin, which play important roles in wound repair in mollusks^40–42^. The cooperative responses to LPS and notching stimulation of these genes related to immunity and calcium metabolism reveal that they may share some points in molecular regulation pathways.

The question remains: can the hemocytes mediate shell formation independent of mantle tissues? Because SMPs are indispensable in shell formation, hemocytes will not be competent for this job without secreting important SMPs. As a result, most SMPs were absent in hemocytes according to the proteome analysis. It should be noted that the calcification-related proteins found in hemocytes in the present and previous study^6^ are barely SMP in the oyster shells. Additionally, the expression levels of the tested SMPs in hemocytes were extremely low (Fig. 6). Although mantle gene 11 was first identified from mantle, it was found to be enriched in hemocytes in the present study. Because its exact role in biomineralization is not known, nor is it present in the shells^43^, we speculated that mantle gene 11 participates in biomineralization indirectly, rather than acts as an SMP. Three possibilities might lead to detection of a few SMPs in the proteome. First, the trace amount of SMP from the EPF was included in the hemocyte samples and detected by LC-MS/MS analysis due to its ultra-high sensitivity. Indeed, the discovered SMPs in hemocytes possessed relatively low scores and abundances (Supplementary Table 4). Second, the hemocytes might encapsulate a handful of SMP when phagocytosing microbes in the EPF. Third, some hemocytes (for example the crystal-bearing granulocytes) may express certain SMPs, but the gene expressions were masked by the whole hemocytes population. More studies are needed to elucidate this possibility. However, hemocytes in *P. fucata* could not be the main source of SMPs according to our results and previous studies^17,44^. Therefore we argued that, confined to provide calcium when necessary, hemocytes in oysters were unlikely to regulate the shell formation process solely, which was slightly different from a previous study^5^.

In conclusion, we found that the hemocytes in *P. fucata* play an auxiliary role to the mantle tissue in shell formation. This is accomplished by promoting calcium transportation, and more importantly, by maintaining the homeostasis in the EPS. Nevertheless, some granulocytes could produce intracellular CaCO_3_ crystals, the molecular basis of which may completely differ from what controls the extracellular shell deposition. Further studies are required to elucidate this interesting phenomenon.

## Materials and Methods

### Pearl oyster

Adult oysters of *P. fucata* were purchased from Guangdong Ocean University and kept in artificial sea water (ASW) upon arrival. The temperature was controlled at 20–25 °C, and the animals were fed every week with yeast and Chlamydomonas.

### Morphology of hemocytes in the EPS

For EPF collection, the shell valves of oysters were opened using a shell opening device. The EPF underneath the mantle was withdrawn using a 5-ml syringe and placed on pyrogen-free glass slides. The slides were kept in a humidity chamber at 25 °C for 30 minutes so that the cells could attach to the bottom. After the incubation, the fluids were removed carefully by pipette and washed twice with marine molluscan balanced salt solution (MMBSS)^45^. Simultaneously, hemocytes from the adductor muscle were collected for comparison. The above-mentioned samples were fixed with 4% paraformaldehyde at 4 °C for 30 minutes and washed three times with MMBSS. Hematoxylin and eosin staining was performed according to the instruction manual (Beyotime Institute of Biotechnology, Haimen, China). Then, the cells were photographed using a Nikon microscope (Nikon Ti-U, Tokyo, Japan).

### Tracking hemocytes in the EPS

Hemocytes extracted from the EPF were mixed with pre-cooled marine anticoagulant solution (MAS, 2.08% sucrose, 0.8% sodium citrate, 0.336% EDTA, 2.23% NaCl, pH 8.0) at a proportion of 1:1 immediately. The pellets were collected by centrifugation at a speed of 500 g for 5 min and resuspended in MAS. A green living-cell fluorescent dye carboxyfluorescein succinimidyl amino ester (CFSE, BD-Pharmingen), with a maximum excitation at 490 nm, was used to label the hemocytes. The collected cells were labelled with 5 µM CFSE (diluted in MAS) for 10 min and washed twice using MMBSS. The labelled cells were adjusted to 10^6^/ml.

For labelled hemocyte injection, 12 recipient individuals were anaesthetized in 0.2% 1-phenoxy-2-propanol (Sigma-Aldrich) dissolved in sea-water. When the valves opened, 50 µl of labelled hemocyte suspension was injected into the adductor muscle or EPS (six individuals for each). The oysters were left for 10 min before returning to the tank. After 24 hours, body fluids from the adductor muscle and EPS were extracted and examined by a Nikon fluorescence microscope.

### Proteomics of total cellular proteins in EPS hemocytes

The EPF from 30 individuals was collected, yielding approximately 10 ml bulk. Cell pellets were obtained by centrifugation and lysed in 0.5 ml of RIPA (Solarbio) to release the cellular protein. Total cellular protein extract (10 µl) was sampled and analyzed by SDS-PAGE (5% stacking gel and 12% running gel). The running process was 100 volts-20 min and then 200 volts-40 min. The SDS-PAGE gel was stained with Coomassie Brilliant Blue. For comparison, the cellular extract of hemocytes from adductor muscle was also sampled. The lane corresponding to the total cellular proteins in the EPF was cut and analyzed by LC-MS/MS as previous described^6^. With no complete genome presently available, we used the catalogue of Pteriidae in UniProtKB and a predicted proteome draft of *P. fucata*^12^ as the searching database.

### Phagocytosis of yeast

Saccharomyces cerevisiae harboring a YFP gene was cultivated in the corresponding culture medium at 28 °C. The yeasts were collected by centrifugation, washed twice in MMBSS, and adjusted to 10^7^/ml. A yeast suspension of 50 µl was injected into the EPS of the anaesthetized oyster. Twelve hours after returning to the tank, EPF was withdrawn to examine phagocytosis. Hemolymph from the adductor muscle was also examined. To rule out that the free yeast cells co-localized with hemocytes randomly, the supernatant was removed after the hemocyte monolayer formed. The cells were washed three times with MMBSS and photographed using an Olympus fluorescence microscope (Japan, IX81-ZDC).

To measure the efficiency of phagocytosis, the YFP-yeast (100 μl, with a concentration of approximately 10^7^ cells/ml) was injected into the EPS of pre-anaesthetized oysters, and then the oysters were returned to the tank. At different time points post injection, the EPF from the oysters were collected for residual yeasts counting.

### Double stain and live cell imaging

The hemocytes were double stained with Calcein-AM (ThermoFisher Scientific) for calcium and DiI (Life Technologies) for the cell membrane. Briefly, hemocytes extracted from the EPS were cultured in a small Petri dish with a glass bottom for 30 min. The fluid was carefully replaced by MMBSS containing 5 μM Calcein-AM and 2.5 μM DiI. The dish was placed in the dark for 30 min at room temperature and washed with MMBSS twice. Then, the hemocytes were cultured with marine bivalves cell culture medium Pf-CM2.5^46^ and photographed using a DeltaVision Elite imaging system (GE, USA). In total, 30 fields of vision were selected and 180 photos were filmed with a time interval of 120 seconds. Three channels were applied (488 nm, 542 nm and polar light). The polar light was used to visualize crystals inside the cells.

### Stimulation of lipopolysaccharide and induction of shell regeneration

Lipopolysaccharide (LPS, InvivoGen) stock was diluted to 5 µg/ml with MMBSS. To mimic immune stimulation in the EPS, 120 adult oysters were divided into four groups spontaneously and anaesthetized as described above. In the first three groups, 50 µl of diluted LPS was carefully injected into the right side of the EPS so as not to injure the mantle. The treated oysters were left for 10 minutes before returning to the tank. The EPF of each group was collected in a 15-ml Eppendorf tube at 0, 12 and 24 hours after incubation and the EPF from each ten individuals was merged to make a biological replicate. In the last group, MMBSS (50 µl) was injected as a negative control and the EPF was collected after 12 hours. The cell pellets were collected by centrifugation at 500 g for 10 min and stored at −80 °C before RNA extraction.

As a comparison, separated experiment was conducted to test gene expressions in the hemocytes from the circulatory system post LPS stimulation. The experimental design was the same with that mentioned above, with the exception that the hemolymph in the adductor muscle sinuses was withdrawn using a 1-ml-syringe.

For the induction of shell regeneration, 120 individuals were collected and 90 oysters were notched at the edges near the middle of the ventral side to make a “V” nick. The last 30 unprocessed oysters were treated as the control group. At 24, 48 and 96 hours post notching treatment, 30 oysters were collect for hemocyte sampling from the EPS as previously described in LPS stimulation.

### RNA extraction and real-time PCR

The collected hemocyte pellets and the mantle tissue (including the central part and the edge) were sampled for RNA extraction using TRIzol reagent (Invitrogen, Carlsbad, CA, USA) as described^44^. The concentration and quality of the purified RNA were determined by a Nanodrop 2000 spectrophotometer (Thermo Scientific, USA) and denaturing RNA electrophoresis in TAE agarose gels. Reverse transcription and RT-PCR were accomplished according to the user manual of PrimeScript™ RT Master Mix (TaKaRa, Shiga, Japan) and SYBR® Premix Ex Taq™ product (TaKaRa, Shiga, Japan), respectively. The primers used are listed in Supplementary Table 1. GADPH was used as an internal reference. Thermal cycles were 94 °C for 30 s, 55 °C for 5 s and 72 °C for 30 s of 40 cycles, with a pre-denature of 94 °C for 5 min by the StepOnePlus™ real-time PCR system (Applied Biosystems, Vernon, CA, USA).

### Statistical analyses

The resulting data were analyzed by SPSS and Excel version 2016. The figures were drawn using SigmaPlot version 11.0 (Systat Software Inc., Germany) and Photoshop CS6 (Adobe, USA). The significance differences between groups were calculated by using one-way analysis of variance (ANOVA).

### Ethics statement

We confirm that this study was approved by the Animal Ethics Committee of Tsinghua University, Beijing, China. All experiments were performed in accordance with relevant guidelines and regulations.

## Electronic supplementary material

Supplementary Information
