# Supplementary material for: Hemocytes in the extrapallial space of Pinctada fucata are involved in immunity and biomineralization
Source: Sci Rep. 2018 Mar 15;8:4657. doi: 10.1038/s41598-018-22961-y (PMC5854705; doi:10.1038/s41598-018-22961-y)

Supplementary information

Hemocytes in the extrapallial space of *Pinctada fucata* are involved in immunity and biomineralization

Jingliang Huang[a], Shiguo Li[a], Yangjia Liu[a], Chuang Liu[ab], Liping Xie[a] & Rongqing Zhang[ac]*

a MOE Key Laboratory of Protein Sciences, School of Life Sciences, Tsinghua University, Beijing 100084, China

b Tsinghua-Peking Joint Center for Life Sciences, School of Life Sciences, Tsinghua University, Beijing 100084, China

c Department of Biotechnology and Biomedicine, Yangtze Delta Region Institute of Tsinghua University, Jiaxing 314000, China

* Corresponding author.

E-mail address: rqzhang@mail.tsinghua.edu.cn (R. Zhang).

Supplementary table 1. Primers used in RT-PCR.

GADPH-F	5' GTGGGAAATCTATCACCGTGC 3'
GADPH-R	5' TGGAGCTAAACAGTTTGTGGTG 3'
DUOX-2-F	5' TACTTAACAAACAAGAGGGCGC 3'
DUOX-2-R	5' CACTGAATTGTCTCGTCCTTGA 3'
ALMP-F	5' GCCAACAGACTAACGTTACC 3'
ALMP-R	5' GTATATTTCTCCCATTCACGC 3'
SOD-F	5' AGGTCTAGCTCCTGGTCAACA 3'
SOD -R	5' CTTGGAGCACCATGCGTTT 3'
Peroxiredoxin-F	5' CAGGGTGGTTTAGGGAACATG 3'
Peroxiredoxin-R	5' ACCCCTGAATGCAATACCCT 3'
galectin-F	5' AGGTGGCGACATTGCACTC 3'
galectin-R	5' CGGCTCCTGATGATAAGAAC 3'
AIF-1-F	5' GAACAAGAAGCCATACTAGATG 3'
AIF-1-R	5' ATGAATAGTACCCGAGTCGG 3'
macroglobulin-F	5' GTCACCTGGTGACCGTATCC 3'
macroglobulin-R	5' CCGTCAGGTTAGCTGTGAAG 3'
mantle gene 11-F	5' GTCTGTCAGGAGTTACCGTT 3'
mantle gene 11-R	5' ACTCTACCGCTGGTTGACTG 3'
MSI31-F	5' GGTAATCTCAACAGCACTCAC 3'
MSI31-R	5' GAACCCAAGAACCTAAGGAA 3'
carbon anhydrase-F	5' CTACACAACATCTCGCGAG 3'
carbon anhydrase-R	5' CCCTAGAGCAGTCTCATATGAC 3'
calponin-3-F	5' GCTATAATGGTCCAACATGTGG 3'
calponin-3-R	5' CCCTTCTGACTGGCAAACTT 3'
calreculin-F	5' GCTGGAAAGTTTTATGGGG 3'
calreculin-R	5' CAGGCCCAAACATGATAAG 3'
Nacrein-F	5' GAGCCAGAGGATGGGGAAA 3'
Nacrein-R	5' GCCTCCATAGGTGAAACGA 3'
KRMP3-F	5' TCACCCTTGGGATTGGAAATGCA 3'
KRMP3-R	5' GCCAAAGTTGTAATCATCGCCACC 3'
MSI60-F	5' GAATGGGACTGGGATGATGACGG 3'
MSI60-R	5' CAATCCAAGTCCGAGTCCAGCAC 3'
ACCBP-F	5' GACATGGAACAAAGATGGTGGA 3'
ACCBP-R	5' CTGTGGCTGGAATGGTTGG 3'
HSP70-F	5' GAGAGGGCAAAGCGAACCT 3'
HSP70-R	5' CGGCGTTCAACTCCTCAAA 3'
camodulin-F	5' TTAGCTTGTTTGACAAGGATGG 3'
camodulin -R	5' TGATCATATCCTGGAGTTCAGC 3'
calponin 2-F	5' TGACTCCCAGAAGGGCATGA 3'
calponin 2-R	5' CCGTCATTCCTTTCTGGCT 3'
Pif80-F	5' CAGAAGTGAAAGATCTGATGGATG 3'
Pif80-R	5' TTTGCCACCTGATACTTTGG 3'

Supplementary table 2. Proteins related to cellular immunity in the proteome of hemocytes from the EPS in *P. fucata*.

Accession	Description	Score	MW [kDa]	calc. pI
CL410.Contig2_All	373 8577 Filamin-C [Crassostrea gigas]	5918.93	291.7	5.90
C1KC80	Galectin OS=Pinctada fucata PE=2 SV=1 - [C1KC80_PINFU]	1090.78	63.4	5.08
M5AKT7	Tropomyosin-1 (Fragment) OS=Pinctada fucata GN=tm-1 PE=2 SV=1 - [M5AKT7_PINFU]	768.31	24.3	4.53
M5AJN8	Tropomyosin-2 (Fragment) OS=Pinctada fucata GN=tm-2 PE=2 SV=1 - [M5AJN8_PINFU]	641.66	24.3	4.54
M5B264	Paramyosin-1 (Fragment) OS=Pinctada fucata GN=pm-1 PE=2 SV=1 - [M5B264_PINFU]	465.70	28.3	5.40
Q3YL64	Transgelin OS=Pinctada fucata PE=2 SV=1 - [Q3YL64_PINFU]	463.98	20.9	8.51
G9JKY4	Integrin beta OS=Pinctada fucata PE=2 SV=1 - [G9JKY4_PINFU]	438.16	88.2	4.96
H2BQ11	Allograft inflammatory factor-1 OS=Pinctada martensii GN=AIF-1 PE=2 SV=1 - [H2BQ11_PINMT]	374.42	17.3	5.15
K1QFI3	Apoptosis-inducing factor 3 [Crassostrea gigas]	341.21	84.9	4.82
CL15482.Contig1_All	minus strand PREDICTED: apoptosis-inducing factor 1, mitochondrial [Strongylocentrotus purpuratus]	239.39	55.4	6.68
C1KBI9	Rab7-like protein OS=Pinctada martensii PE=2 SV=1 - [C1KBI9_PINMT]	214.01	23.1	5.48
B5MCX3	Septin-2 OS=Homo sapiens GN=SEPT2 PE=2 SV=1 - [B5MCX3_HUMAN]	199.47	36.9	6.18
A0A0D5CBX6	C-type lectin-1 OS=Pinctada martensii PE=2 SV=1 - [A0A0D5CBX6_PINMT]	149.05	17.6	7.71
CL3266.Contig2_All	Macrophage asialoglycoprotein-binding protein 1 [Crassostrea gigas]	105.35	16.1	7.68
Unigene14090_All	minus strand TNF receptor-associated factor 2 [Crassostrea gigas]	88.94	31.0	7.34
CL2458.Contig2_All	Caspase-7 [Crassostrea gigas]	84.02	33.0	6.01
F8RTT9	F-type lectin OS=Pinctada martensii PE=2 SV=1 - [F8RTT9_PINMT]	33.16	21.4	8.18
M5AKK3	Troponin-T (Fragment) OS=Pinctada fucata GN=tn-T PE=2 SV=1 - [M5AKK3_PINFU]	27.17	19.2	5.44
A0A0B5FZ77	Cyclophilin B OS=Pinctada martensii PE=2 SV=1 - [A0A0B5FZ77_PINMT]	25.53	24.8	6.14
I7D6W0	F-type lectin 1 OS=Pinctada fucata PE=2 SV=2 - [I7D6W0_PINFU]	22.37	21.7	8.16
D0EFL7	Interferon-gamma-inducible lysosomal thiol reductase OS=Pinctada fucata PE=2 SV=1 - [D0EFL7_PINFU]	14.11	28.9	7.40
M5B265	Twitchin-3 (Fragment) OS=Pinctada fucata GN=tw-3 PE=2 SV=1 - [M5B265_PINFU]	7.78	23.1	4.54
A9P668	NF-kappa B OS=Pinctada fucata PE=2 SV=1 - [A9P668_PINFU]	7.69	71.6	5.90

Supplementary table 3. Proteins related to humoral immunity and peptidases in the proteome of hemocytes from the EPS in *P. fucata*.

Accession	Description	Score	MW [kDa]	calc. pI
L8AZW8	Copper/zinc superoxide dismutase (Fragment) OS=Pinctada fucata GN=sod-1 PE=2 SV=1 - [L8AZW8_PINFU]	324.38	8.4	6.01
K9L8I5	Cathepsin D OS=Pinctada margaritifera PE=2 SV=1 - [K9L8I5_PINMG]	302.51	42.2	6.55
E9NS22	Catalase OS=Pinctada fucata PE=2 SV=1 - [E9NS22_PINFU]	270.70	58.0	8.41
F8RNZ7	Cathepsin D OS=Pteria penguin PE=2 SV=1 - [F8RNZ7_PTEPN]	232.72	42.3	8.03
S5S578	Superoxide dismutase OS=Pinctada fucata PE=3 SV=1 - [S5S578_PINFU]	232.36	20.4	6.92
ADE45341	77 4231 minus strand thioester-containing protein 1.4 [Biomphalaria glabrata]	229.19	154.3	7.03
Unigene14680_All	576 4805 Dual oxidase 2, partial [Crassostrea gigas]	199.07	161.3	7.65
Q2VU37	Metalloendopeptidase OS=Pinctada fucata PE=2 SV=1 - [Q2VU37_PINFU]	187.82	78.1	7.88
A0A0K1RMV1	Peroxiredoxin OS=Pinctada fucata PE=4 SV=1 - [A0A0K1RMV1_PINFU]	157.23	22.3	7.77
CL7350.Contig2_All	279 1658 bactericidal permeability increasing protein [Crassostrea gigas]	134.16	50.5	9.26
XP_003389946	PREDICTED: fibrinogen C domain-containing protein 1-A-like [Amphimedon queenslandica]	127.42	24.1	6.11
Q7YW83	Ferritin OS=Pinctada fucata PE=2 SV=1 - [Q7YW83_PINFU]	121.32	23.6	5.96
A0A0C4Y3Z7	Vitellogenin-6 mRNA (Fragment) OS=Pinctada margaritifera PE=2 SV=1 - [A0A0C4Y3Z7_PINMG]	108.14	61.6	9.16
Unigene22461_All	121 1551 Glutaredoxin [Crassostrea gigas]	102.41	54.6	5.91
F1DH40	Cathepsin B OS=Pinctada fucata PE=2 SV=1 - [F1DH40_PINFU]	85.84	40.8	7.09
CL1957.Contig2_All	152 751 glutathione S-transferase pi [Mytilus edulis]	66.47	22.9	7.03
D1G103	Cathepsin L OS=Pinctada fucata PE=2 SV=1 - [D1G103_PINFU]	64.27	40.5	5.40
A0A0K0L8X1	Alpha-2-macroglobulin OS=Pinctada fucata PE=2 SV=1 - [A0A0K0L8X1_PINFU]	31.04	223.0	6.25
A0A0E3DAN0	Cytochrome P450 family 4 OS=Pinctada martensii GN=CYP4 PE=2 SV=1 - [A0A0E3DAN0_PINMT]	33.92	58.0	7.96
S5FPE0	Metallothionein 2 OS=Pinctada martensii PE=2 SV=1 - [S5FPE0_PINMT]	10.70	7.7	7.17
A0A0B4ZY93	Granulin epithelin variant 2 OS=Pinctada fucata GN=GEP PE=2 SV=1 - [A0A0B4ZY93_PINFU]	9.01	156.1	7.06
I6TCY8	Superoxide dismutase [Cu-Zn] OS=Pinctada fucata GN=Cu PE=2 SV=1 - [I6TCY8_PINFU]	8.15	15.9	6.32

Supplementary table 4. Proteins related to calcification in the proteome of hemocytes from the EPS in *P. fucata*.

Accession	Description	Score	MW [kDa]	calc. pI
B2KKR0	Calcium-transporting ATPase OS=Pinctada fucata GN=SERCA PE=2 SV=1 - [B2KKR0_PINFU]	1350.39	111.2	5.73
A6YIE3	Calreticulin OS=Pinctada fucata PE=2 SV=1 - [A6YIE3_PINFU]	1170.37	48.0	4.64
A0A0K1RNC3	N-U8 OS=Pinctada fucata PE=4 SV=1 - [A0A0K1RNC3_PINFU]	1172.43	56.9	8.76
M5AJP3	Calponin-3 (Fragment) OS=Pinctada fucata GN=cp-3 PE=2 SV=1 - [M5AJP3_PINFU]	621.53	31.9	6.90
M5AKU2	Calponin-2 (Fragment) OS=Pinctada fucata GN=cp-2 PE=2 SV=1 - [M5AKU2_PINFU]	228.80	21.4	8.28
Q6EEV2	Calmodulin OS=Pinctada fucata PE=2 SV=1 - [Q6EEV2_PINFU]	164.57	16.9	4.27
E5RQ31	Carbonic anhydrase-related protein VIII OS=Pinctada fucata GN=CARP VIII PE=2 SV=1 - [E5RQ31_PINFU]	155.21	29.2	6.90
A9P669	Calcium-transporting ATPase OS=Pinctada fucata PE=2 SV=1 - [A9P669_PINFU]	56.07	130.7	6.14
Q45TJ9	Mantle protein 11 OS=Pinctada fucata GN=MG11 PE=2 SV=1 - [Q45TJ9_PINFU]	49.94	22.5	9.38
C1ITK0	Calcineurin B subunit OS=Pinctada fucata PE=2 SV=1 - [C1ITK0_PINFU]	43.28	19.2	4.79
A6XBS1	Amorphous calcium carbonate binding protein 1 OS=Pinctada fucata PE=2 SV=1 - [A6XBS1_PINFU]	39.12	27.1	4.93
Q3YL62	Mantle gene 4 OS=Pinctada fucata PE=2 SV=1 - [Q3YL62_PINFU]	37.38	17.9	8.02
Q3BDI8	Calmodulin-like protein OS=Pinctada fucata PE=2 SV=1 - [Q3BDI8_PINFU]	24.53	18.4	4.15
P86947	Valine-rich protein OS=Pinctada maxima PE=1 SV=1 - [VRP_PINMA]	19.57	27.1	10.93
U3G0C6	Pif OS=Pinctada maxima GN=pif PE=2 SV=1 - [U3G0C6_PINMA]	11.80	110.7	5.08

Supplementary table 5. Real time PCR data of selected genes show the relative gene expression in the mantle tissue and the hemocytes of *P. fucata*.

**Gene**	**Ratio of gene expression level (mantle/ hemocytes)**	**Log_{10} (M/H)**
KRMP-3	169865	5.230104
nacrein	17583	4.245093
ACCBP	4734	3.675228
MSI31	511	2.708421
MSI60	184	2.264818
HSP70	9.560456	0.980479
Pif80	7.686692	0.885739
galectin	0.951848	-0.02143
macroglobulin	0.7842	-0.10557
Mantle gene 11	0.054492	-1.26367
SOD	0.047096	-1.32701

Supplementary figure 1, SDS-PAGE of total cellular proteins in hemocytes from the EPS and adductor muscle. Lane EPF, hemocytes from the EPS; lane AMS, hemocytes from adductor muscle; M, protein marker.

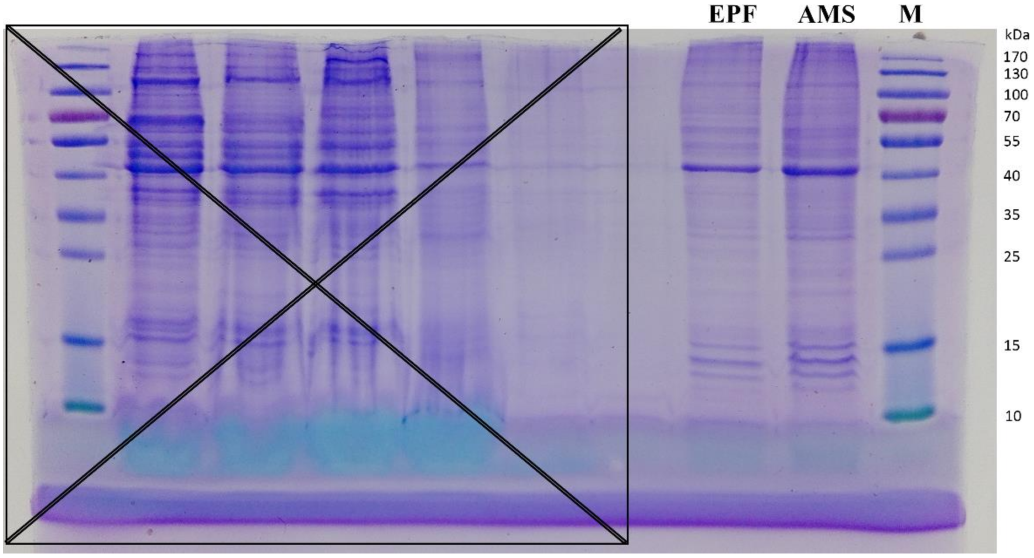

Supplementary figure 2. Quantity measurement of microbes in the hemolymph and EPF from *P. fucata* and the ambient environment. The hemolymph and EPF were withdrawn freshly and inoculated immediately onto 2216 marine microbe culture plates. In total 50μl of each sample was spread on the plates, and each column represents the mean of three repeats.

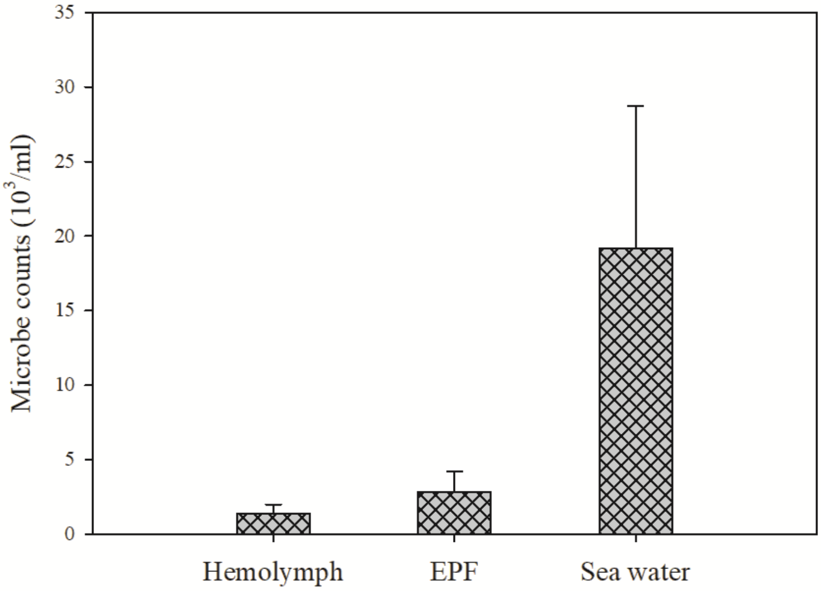

Supplementary figure 3. Gene expression of hemocytes in the adductor muscle sinuses after stimulation with LPS. Genes involved in both immunity and the calcification process were determined by RT-PCR. ALMP, astacin-like metalloproteinase; DUOX-2, dual oxidase 2; AIF, Allograft inflammatory factor; CA, carbonic anhydrase-related protein VIII. ($P < 0.05$)

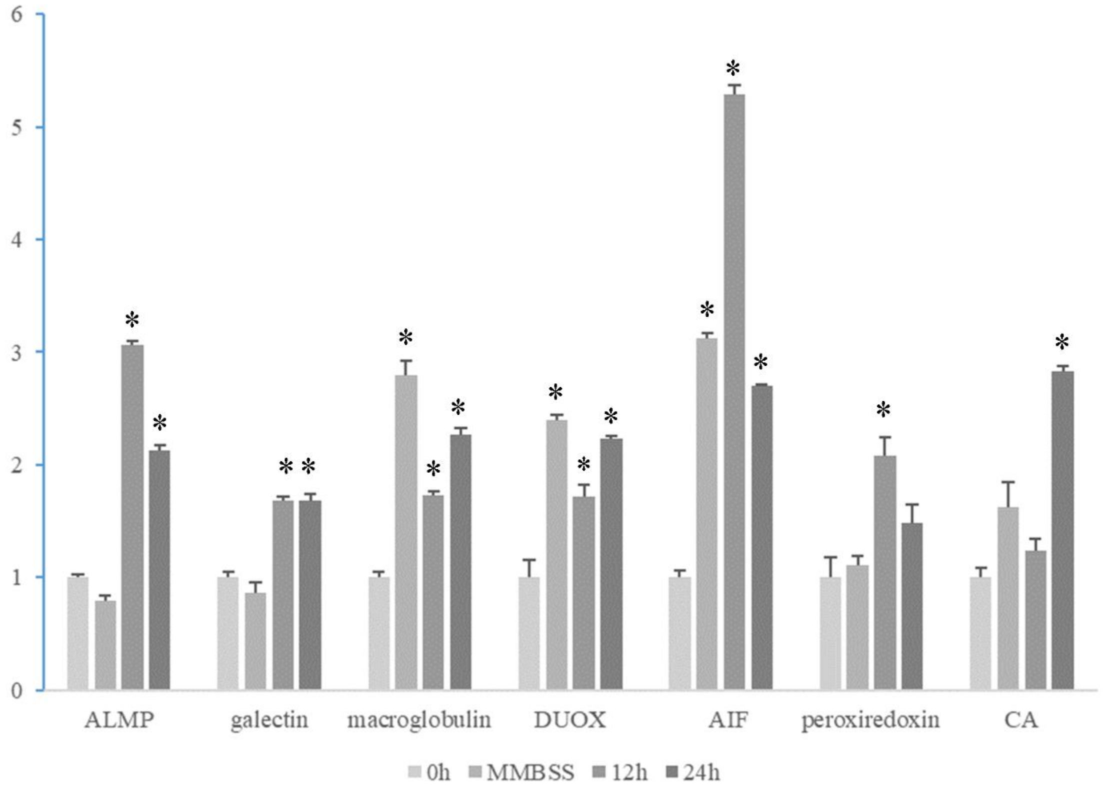

Supplement: Supplementary file 1 — Supplementary Information [file 41598_2018_22961_MOESM1_ESM.pdf]
